# A multicentre evaluation exploring the impact of an integrated health and social care intervention for the caregivers of ICU survivors

**DOI:** 10.1186/s13054-022-04014-z

**Published:** 2022-05-24

**Authors:** Joanne McPeake, Philip Henderson, Pamela MacTavish, Helen Devine, Malcolm Daniel, Phil Lucie, Lynn Bollan, Lucy Hogg, Mike MacMahon, Sharon Mulhern, Pauline Murray, Laura O’Neill, Laura Strachan, Theodore J. Iwashyna, Martin Shaw, Tara Quasim

**Affiliations:** 1 Intensive Care Unit, Glasgow Royal Infirmary, Glasgow, UK; 2 School of Medicine, Dentistry and Nursing, University of Glasgow, Glasgow, UK; 3 Intensive Care Unit, University Hospital Wishaw, Wishaw, UK; 4 Intensive Care Unit, NHS Fife, Kirkcaldy, UK; 5 Intensive Care Unit, NHS Ayrshire and Arran, Kilmarnock, UK; 6 Intensive Care Unit, University Hospital Monklands, Airdrie, UK; 7 Intensive Care Unit, Queen Elizabeth University Hospital, Glasgow, UK; 8 Center for Clinical Management Research, VA Ann Arbor Health System, Ann Arbor, MI USA; 9 Division of Pulmonary and Critical Care, Department of Internal Medicine, University of Michigan, Ann Arbor, MI USA; 10 Clinical Physics, NHS Greater Glasgow and Clyde, Glasgow, UK

**Keywords:** Critical care, Rehabilitation, Family, Caregivers and mental health

## Abstract

**Background:**

Caregivers and family members of Intensive Care Unit (ICU) survivors can face emotional problems following patient discharge from hospital. We aimed to evaluate the impact of a multi-centre integrated health and social care intervention, on caregiver and family member outcomes.

**Methods:**

This study evaluated the impact of the Intensive Care Syndrome: Promoting Independence and Return to Employment (InS:PIRE) programme across 9 sites in Scotland. InS:PIRE is an integrated health and social care intervention. We compared caregivers who attended this programme with a contemporary control group of ICU caregivers (usual care cohort), who did not attend.

**Results:**

The primary outcome was anxiety measured via the Hospital Anxiety and Depression Scale at 12 months post-hospital discharge. Secondary outcome measures included depression, carer strain and clinical insomnia. A total of 170 caregivers had data available at 12 months for inclusion in this study; 81 caregivers attended the InS:PIRE intervention and completed outcome measures at 12 months post-hospital discharge. In the usual care cohort of caregivers, 89 completed measures. The two cohorts had similar baseline demographics. After adjustment, those caregivers who attended InS:PIRE demonstrated a significant improvement in symptoms of anxiety (OR: 0.42, 95% CI: 0.20–0.89, *p* = 0.02), carer strain (OR: 0.39; 95% CI: 0.16–0.98 *p* = 0.04) and clinical insomnia (OR: 0.40; 95% CI: 0.17–0.77 *p* < 0.001). There was no significant difference in symptoms of depression at 12 months.

**Conclusions:**

This multicentre evaluation has shown that caregivers who attended an integrated health and social care intervention reported improved emotional health and less symptoms of insomnia, 12 months after the delivery of the intervention.

**Supplementary Information:**

The online version contains supplementary material available at 10.1186/s13054-022-04014-z.

## Background

The profile of Intensive Care Unit (ICU) survivorship has been brought into sharp focus due to the COVID-19 pandemic [1, 2]. Following a critical illness, patients can experience clinically important physical, emotional and cognitive symptoms [3–5]. These problems can have major implications for the patient and healthcare system [6, 7].

Less focus has been placed on family members and caregivers of ICU survivors. This vulnerable group often have a challenging trajectory during the critical care recovery period and are known to experience emotional and social problems [8–10]. For example, recent evidence has shown that over two thirds of caregivers may experience symptoms of depression, up to 80% can experience symptoms of anxiety and more than half can experience caregiver strain in the months following critical care discharge [11–13].

To address this pressing issue, we designed a multi-centre health and social care intervention to support caregivers recovery following critical care discharge. Specifically, we sought to understand what impact this intervention had on emotional outcomes and social disruption for the caregiver.

## Methods

### Study design

Using a multicentre cohort design, we compared caregivers who attended an integrated health and social care intervention aimed at improving outcomes for ICU survivors, with a contemporary, usual care cohort (caregivers who had been exposed to critical care and were currently caring for a critical care survivor) who did not attend the programme. Previous work has described the outcomes of patients who participated in this intervention, alongside a detailed process evaluation [14].

A *caregiver* was defined as the individual who provided most of the financial, emotional and physical support for the patient, or the individual primarily responsible for caring for the patient on an unpaid basis [15].

We report a cohort study, as per the Strengthening the Reporting of Observational Studies in Epidemiology (STROBE) guidelines [16].

### Approval

Ethical approval was granted by the Northwest (Liverpool Central) Research Ethics Committee (reference number: 17/NM/0199). All participants provided written consent.

### Intervention

The intervention is described in accordance with the Template for intervention Description and Replication checklist [17] (Additional file 1: S1).

The Intensive Care Syndrome: Promoting Independence and Return to Employment (InS:PIRE) programme is a complex intervention and has been described previously [18–20]. InS:PIRE is an integrated health and social care intervention aimed at improving outcomes for ICU survivors and their caregivers in the year following hospital discharge.

Briefly, all patients receive individual reviews with different members of the multidisciplinary team (MDT) including: an ICU nurse and doctor; pharmacist; and physiotherapist. These specialists offer a debrief of the ICU stay, an assessment of ongoing problems, goal setting and patient-directed management plans. Clinical Psychology services, alongside occupational therapy, was also available. Peer support was embedded throughout InS:PIRE through the use shared waiting areas and group sessions. Patient and caregiver volunteers further along the recovery trajectory also attended the programme and provided peer support [21].

Several group sessions were set up for caregivers, including the clinical psychological support. Every site involved local community organisations who provided welfare advice including information on relevant government benefits and housing. This support was delivered through a combination of individual appointments, drop-in sessions or group discussions. Support for caregivers from local agencies was also integrated at every site. In contrast with other ICU recovery services which often include a single clinical appointment, InS:PIRE was designed as a recovery programme; patients and caregivers attend each week for five weeks and are followed up for 1 year after initial attendance. During the intervention period, the InS:PIRE service took place in the hospital setting.

InS:PIRE was co-designed with local patients and caregivers. During this process, patients and caregivers both described the necessity for a specific and deliberate focus on caregivers within any rehabilitation service. This is also consistent with previous evidence is this field [22]. Initial single-centre work showed that supporting caregivers was feasible, safe and acceptable; thus, this support was included in this multi-centre scale up [12, 23]. Caregivers could attend all sessions and were eligible to receive the same support as patients. This only differed with the physiotherapy and pharmacy sessions which were aimed specifically at patient recovery.

### Participants

Participants were invited to the programme between 4 and 12 weeks after hospital discharge. Patient inclusion criteria were any patient receiving level three care (multiple organ support and / or invasive respiratory support) or more than seven days of level two care (single organ support or postoperative care). There was no upper age limit for inclusion. Exclusion criteria were any patient who was terminally ill, had suffered a traumatic brain injury or was an in-patient under psychiatric services. Caregiver inclusion criteria were: paired consented patient data had to be available and caregiving responsibilities occurred in an informal, non-paid basis. We limited inclusion in the research to one caregiver per patient; however, multiple caregivers could attend InS:PIRE.

### Intervention cohort

Five sites provided the InS:PIRE programme as part of a quality improvement collaborative (*intervention cohort*) over 2 years. Caregivers in the intervention cohort were consecutively recruited to this study between May 2016 and October 2018 (follow-up completed December 2019). Caregiver outcome measures were completed via a pre-planned 12-month follow-up appointment. Participants were given the opportunity to complete questionnaires at InS:PIRE attendance or via telephone. Fidelity of the intervention was assessed by the number of patients who completed the three ‘core’ interventions: medical/nursing consultation; pharmacy; and physiotherapy review. As such, only the caregivers of patients who completed these core interventions were included in this final analysis.

### Usual care cohort

We recruited a contemporary usual care cohort from sites who had no active follow-up or rehabilitation for ICU survivors. Patients in the usual care cohort were recruited from eight hospitals in Scotland between 10 and 16 months post-hospital discharge. Patients were identified by searching the local electronic records system and were recruited by postal survey. Questionnaire packs were sent to eligible patients with pre-paid return envelopes. Alongside these patient packs, we asked the patient’s primary caregiver (or closest family member) to complete a separate pack. Reminder questionnaire packs were sent if the pack was not returned after one month. Participants were also given opportunity to call to discuss issues or recruitment with researchers. The usual care cohort was recruited between July 2017 and March 2020. Ethical approval was in place to continue beyond March 2020; however, the impact of the coronavirus disease 2019 pandemic was unknown; thus, recruitment to this study was closed in an attempt to reduce any confounding effect.

### Data collection

We created a short questionnaire in order to collect family member demographics. Data collected included age, relationship to patient and gender.

In-hospital patient demographic and clinical data were obtained from clinical notes and discharge summaries. Patient comorbidity data (including mental health data) were obtained from medical notes and critical care admission records. Critical care length of stay was taken for the highest level of care and during the first critical care admission only.

The Scottish Index of Multiple Deprivation (SIMD) is produced by the Scottish Government as a measure of deprivation, with postcode areas defining data on socio-economic status. This research split the SIMD into five categories to define socio-economic status; quintile one represented the most deprived and quintile five the least [24].

### Outcome measures

#### Hospital Anxiety and Depression Scale

The primary outcome measure in this study was caregiver anxiety. Anxiety and depression were measured using the Hospital Anxiety and Depression Score (HADS) [25]. The HADS questionnaire contains 14 statements relating to mood, with seven statements relating to depression and seven to anxiety. Each statement has four potential options (scored 0–3). The cutoff points widely adopted for HADS are described in Additional file 2: S2. In this analysis, we classified anxiety and depression as a score of 8 or above utilising the score subscales [25]. Appropriate licensing requirements were in place for the use of the HADS.

#### Carer Strain Index

Alongside the HADS, the Carer Strain Index (CSI) was utilised [26]. The CSI measures strain, including social strain, related to care provision from the caregiver’s perspective. There are elements related to emotional adjustment, social issues, physical and financial strain. Each question is given one point; a score of seven or greater is the generally accepted cut off point for a high level of strain.

#### The Insomnia Severity Index

The Insomnia Severity Index (ISI) is a seven-item questionnaire which has been validated as a screening tool for clinical insomnia. Participants are asked to rank the severity of their sleep problems on a scale of zero to four and answer four other questions regarding satisfaction with their sleeping patterns [27]. We defined insomnia as a score of 8 and greater from a maximum score of 28 (range 0 to 28).

### Statistical analysis

Analyses were undertaken using R (Version 4.0.3). All missing covariates were imputed using categorical and regression trees analysis with the Multivariate Imputation by Chained Equations (MICE) software package. We used 5 imputations and 10 iterations.

Continuous variables were expressed as medians and interquartile range (IQR). The Kruskall–Wallis test was used to compare different sub-groups and the Chi-squared test to analyse categorical variables. Logistic regression was used to understand the impact of the intervention on the main outcomes of this study.

Models were created using domain knowledge and previous evidence in the field. All models were adjusted for: relationship with the patient; caregiver age; caregiver gender; time to follow-up; and socio-economic status (SIMD quintiles). We also adjusted patient level demographics which are known to influence recovery including: hospital length of stay; patient age; and the presence of a mental health comorbidity (pre-existing).

## Results

### Demographics

Two hundred and six patients attended InS:PIRE across the five participating sites and consented to participation in the study. Of these patients, 136 attended with caregivers who consented to participation in the study at baseline (initial InS:PIRE attendance). Eighty-one (60%) of these caregivers completed outcomes measures at 1 year and were eligible for inclusion in this analysis (*intervention cohort*).

In the usual care cohort, 452 patients were sent questionnaire packs across the four usual care cohort sites; 115 patients returned the packs for analysis in the study. Of these, 89 (77%) paired caregivers also completed outcome measures at 1 year and were included in the caregiver usual care cohort.

The caregiver intervention and usual care cohort was similar in all baseline demographics. Both cohorts were predominantly female (intervention: 49 (60.5%) vs. usual care: 57 (64%)) and had similar age profiles (intervention:58 (IQR: 48.0–66.3) vs. usual care 58 (IQR: 47.7–69.0) years (Table 1). There was also a similar spread across the socio-economic gradient in both groups. Patient and caregiver demographics are shown in Table 1.

**Table 1:** Caregiver demographics, alongside paired patient demographics

	Usual care cohort (*n* = 89)	Intervention cohort (*n* = 81)	*p* value
Caregiver demographics			
Relationship with patient, *n* (%)			0.47
Partner or spouse	66 (74.2)	54 (66.6)	
Child or grandchild	16 (18)	13 (16.1)	
Parent	3 (3.3)	8 (9.9)	
Other	4 (4.5)	6 (7.4)	
Age, years, median (IQR)	58.0 (47.7–69.0)	58.0 (48.0–66.3)	0.88
Gender, male, *n* (%)	32 (36.0)	30 (37.0)	0.79
Socio-economic status			0.50
SIMD 1 (most deprived)	25 (28.1)	20 (24.7)	
SIMD 2	19 (21.3)	19 (23.4)	
SIMD 3	12 (13.5)	16 (19.8)	
SIMD 4	12 (13.5)	7 (8.6)	
SIMD 5 (least deprived)	19 (21.3)	11 (13.6)	
Patient demographics			
Age at ICU admission, median (IQR)	63.9 (49.7–71.4)	58.7 (51.1–67.7)	0.16
Gender, male, number (%)	50 (56.2)	41 (50.6)	0.33
ICU LOS, days, median (IQR)	4.75 (2.4–9.6)	11.3 (7.0–19.7)	< 0.01
Hospital LOS, days, median (IQR)	19.0 (11.2–33.5)	32.0 (17.0–51.7)	< 0.01
APACHE II, median (IQR)	19.0 (14.9–25.0)	19.0 (15.0–26.0)	0.49
Mental health issues pre-ICU, *n* (%)	20 (22.5)	21 (25.9)	0.72
Ventilation required, *n* (%)	76 (85.4)	77 (95.1)	0.12
Two or more comorbidities, *n* (%)	42 (47.2)	34 (42)	0.34
Surgical admission	42 (47.2)	31 (38.3)	0.17
Time to follow-up, median months (IQR)	15.0 (13.1–16.5)	16.0 (14.8–17.5)	< 0.01

Missing data information available in Additional file 2: S2

*IQR* interquartile range, *SIMD* Scottish Index of Multiple Deprivation, *ICU* Intensive Care Unit, *LOS* length of stay, *APACHE II* Acute Physiology and Chronic Health Evaluation

The dataset was 96.2% complete; a breakdown of the missing variables is shown in Table 2.

**Table 2:** Missing values per variable for caregivers (intervention and usual care)

Variable	Missingness, *N* (170) (%)
Caregiver demographics	
Caregiver relationship with patient	2 (1.2)
Caregiver age	24 (14.1)
Caregiver gender	2 (1.2)
Caregiver SIMD	10 (5.9)
Caregiver time to follow-up	6 (3.5)
Hospital anxiety and depression scale (HADS) questions	
I feel tense or wound up	6 (3.5)
I still enjoy the things I used to enjoy	4 (2.4)
I get a sort of frightened feeling as if something awful is about to happen	4 (2.4)
I can laugh and see the funny side of things	3 (1.8)
Worrying thoughts go through my mind	4 (2.4)
I feel cheerful	3 (1.8)
I can sit at ease and feel relaxed	4 (2.4)
I feel as if I am slowed down	4 (2.4)
I get a sort of frightened feeling like “butterflies” in the stomach	5 (2.9)
I have lost interest in my appearance	5 (2.9)
I feel restless as if I have to be on the move	7 (4.1)
I look forward with enjoyment to things	6 (3.5)
I get sudden feelings of panic	5 (2.9)
I can enjoy a good book or radio or television programme	8 (4.7)
Carer strain index questions	
Sleep is disturbed	5 (2.9)
It is inconvenient	6 (3.5)
It is a physical strain	7 (4.1)
It is confining	7 (4.1)
There have been family adjustments	5 (2.9)
There have been changes in personal plan	5 (2.9)
There have been emotional adjustments	7 (4.1)
Some behaviour is upsetting	5 (2.9)
It is upsetting to find has changed so much from his or her former self	10 (5.9)
There have been work adjustments	7 (4.1)
It is a financial strain	5 (2.9)
Feeling completely overwhelmed	5 (2.9)
There have been other demands on my time	5 (2.9)
Insomnia severity index questions	
Difficulty falling asleep	19 (11.2)
Difficulty staying asleep	20 (11.8)
Problem wakening up too early	31 (18.2)
How many nights per week were you bothered by problems sleeping	4 (2.4)
How satisfied or dissatisfied are you with your current sleep pattern	4 (2.4)
To what extent do you consider your sleep problem to interfere with your daily functioning	3 (1.8)
How noticeable to others do you think your sleeping problem is in terms of impairing the quality of your life	3 (1.8)
How worried or distressed are you about your current sleep problem	3 (1.8)

*SIMD* Scottish Index of Multiple Deprivation, *ICU* Intensive Care Unit, *APACHE* Acute Physiology and Chronic Health Evaluation

### Outcomes

#### Anxiety and depression

After adjustment, there was a 58% adjusted odds reduction in symptoms of anxiety (measured as a score of 8 or greater on the HADS anxiety scale) in those caregivers who received the InS:PIRE intervention (OR: 0.42, 95% CI: 0.20–0.89, *p* = 0.02) (Table 3). The number of caregivers who had depression (classified as a score of 8 or greater on the HADS depression scale) was 27% in the usual care cohort versus 22% in the intervention cohort; however, the odds of depressive symptoms at 12 months was not significantly different in those who received the InS:PIRE intervention, (OR: 0.58, 95% CI: 0.26–1.31, *p* = 0.19) (Table 3).

**Table 3:** Summary of the effect of InS:PIRE on study outcomes at 12 months estimated via logistic regression models

Outcome measure	Adjusted estimate	*p* value	95% confidence interval
HADS anxiety	0.42	**0.02**	0.20–0.89
HADS depression	0.58	0.19	0.26–1.31
Carer strain	0.39	**0.04**	0.16–0.98
Clinical insomnia	0.36	**< 0.001**	0.17–0.77

Statistical Signifance was defined as* p* < 0.05

### Carer strain

After adjustment, there was a significant, 61% reduction in the odds of carer strain in those who received the InS:PIRE intervention (OR: 0.39; 95% CI: 0.16–0.98 *p* = 0.04). The rate of strain (defined as a score equal to or greater than 7) was 30% in the usual care cohort compared to a rate of 19% in the intervention group. There were no significant differences between individual components of the CSI. Considering both groups together, 25% of caregivers indicated that there had been work adjustments during the recovery period and over one quarter (26%) described financial strain.

### Insomnia

After adjustment, there was a significant reduction in the odds of clinically important Insomnia, measured via the ISI in those caregivers who received the InS:PIRE intervention by 65% (OR: 0.36; 95% CI: 0.17–0.77 *p* < 0.001) (Table 3). The incidence of insomnia (defined as an ISI score above 7) was 61% in the usual care cohort, and 38% in the intervention group.

**Fig. 1:**
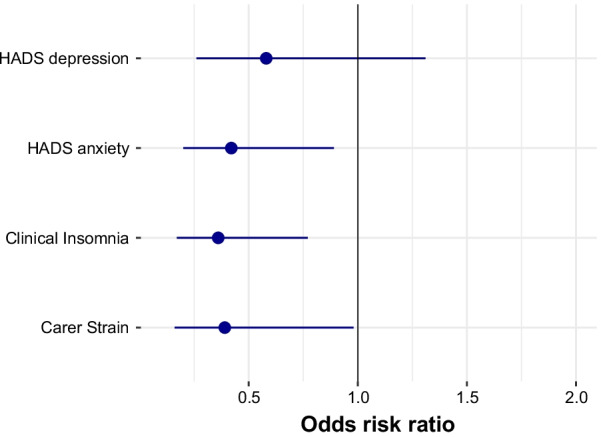
Coefficients estimates demonstrating the impact of the InS:PIRE programme on caregiver outcomes at 1 year. Odds ratio of risk of screening positive for each outcome measure at 1 year in the InS:PIRE intervention cohort compared to the usual care cohort. Estimate with 95% confidence interval. Anxiety and depression: measured by Hospital Anxiety and Depression Scale (HADS), positive screening if score ≥ 8/21 for each component score; Carer strain: measured by Carer Strain Index (CSI) with positive screening defined as a score of ≥ 7/13; Insomnia: measured using the Insomnia Severity Index (ISI) with positive screening defined as a score of ≥ 8/28

A visual representation of the estimated impact of the InS:PIRE programme on caregiver outcomes at 12 months is shown in Fig. 1. A full description of all outcome model estimates is provided in Additional file 3: S3.

## Discussion

This evaluation of a multicentre integrated health and social care intervention demonstrated a positive impact on the emotional health of ICU survivor caregivers at 12 months. As far as we can establish, this is the first multicentre intervention internationally to report benefit for caregivers during the post-ICU recovery phase.

The InS:PIRE intervention was designed as a peer supported, multi-disciplinary programme for both patients and caregivers [19]. Previous research has described multiple benefits of peer support for both patients and caregivers in the post-ICU setting such as reduced anxiety, improved external validation of progress and support with expectation management [28, 29]. A further mechanism which we hypothesise has driven improvements, is the intentional focus on caregiver outcomes, rather than caregiver outcomes being viewed as a biproduct of patient treatment. Intentional use of separate caregiver education sessions alongside the integration of local community services which focused on caregiver needs was available at every site. However, it is important to recognise that targeted treatments for both patients and caregivers are likely to have had a bidirectional effect across the family unit. That is, a patient knowing a caregiver is receiving the support they require will potentially have a positive effect on their recovery, and likewise a caregiver seeing the patient receiving the necessary support to recover will likely reduce anxiety and mental health problems.

Carer strain also significantly reduced for those caregivers who attended the programme. The Carer Strain Index is a tool which examines all the global aspects of caring responsibilities (Additional file 1: S1). This includes domains such as return to employment, physical and financial strain [26]. Caregivers had access to social, financial and welfare advice for the duration of the programme; this may well have been the driver for the reduction in carer strain seen. This finding is consistent with work from primary care in the UK; improving the social and financial situation of people, by co-locating social and welfare services in health centres resulted in improvements in mental health [30]. Moreover, recent evidence has demonstrated a direct correlation between emotional and social outcomes in ICU survivors [31]. Future interventions aimed at addressing outcomes in this group must ensure that all aspects of an individual’s wellbeing are considered.

Although there were less symptoms of depression in the InS:PIRE cohort, the intervention did not significantly improve symptoms of depression for caregivers at 12 months. Previous literature has demonstrated that symptoms such as depression improve over the first year of recovery for most critical care survivors [32]. We hypothesise that caregivers have a different recovery trajectory to patients and thus may require targeted interventions at different time periods. There is limited evidence describing the trajectories of symptoms beyond 2 months for caregivers following exposure to critical care illness [33]. Given the large volume of informal, unfunded care provided by this caregiver group in the months following hospital discharge, it is crucial that health services offer adequate support to this cohort. More research is urgently required in this area, in order to fully understand how best to support this vulnerable group, in relation to symptoms of depression.

### Limitations

Study limitations are notable. Firstly, we do not know if the caregivers included in this study had pre-existing mental health problems. As such, we are unable to account for an important baseline characteristic in our analysis. Secondly, this was not a randomised control trial. While there was substantial overlap in baseline characteristics between the intervention and usual care cohorts, they were not randomly assigned to either usual care or the intervention; as such, this limits the ability to draw casual inference from the results. Moreover, we experienced attrition over the course of the 12 months, common with other studies in the field, which may have also influenced the reported outcomes [34]. We also do not have data on caregivers who attended the InS:PIRE intervention but did not consent to recruitment in this study. Their experience of the programme may be distinctly different from those represented in this analysis.

It could be argued that the patients and caregivers who attended the InS:PIRE programme were more engaged and motivated about improving their health, thus, accounting for the improved outcomes seen in the intervention cohort. However, there was diversity in the cohort across the socio-economic gradient. Moreover, 40% of patients in the intervention cohort had multi-comorbidities and over a quarter had pre-existing mental health issues. This would suggest that the intervention arm did include patients with significant chronic issues, who often have challenging trajectories following critical illness [35].

The method of data collection varied across the two cohorts (in person or telephone vs. postal completion). This could have influenced how the outcomes were recorded and reported for each cohort. Finally, we only have data at one time point for the control cohort. As such we cannot fully understand the symptom trajectory for caregivers, which limits the interpretability of some of the results.

## Conclusions

This multicentre evaluation has shown that caregivers who attended the InS:PIRE programme reported improved emotional health and less symptoms of insomnia 12 months after hospital discharge, in comparison with a contemporary control group. More work is required to understand how the recovery trajectory for this group interacts with the patient recovery trajectory, to ensure services fully support all needs in this group.

## Supplementary Information

**Additional file 1: S1.** The template for intervention description and replication checklist (S1).

**Additional file 2: S2.** Details of Hospital Anxiety and Depression Scale alongside common cut-off points.

**Additional file 3: S3.** Logistic regression model outcomes.

## Data Availability

The datasets used and/or analysed during the current study are available from the corresponding author on reasonable request.

## Abbreviations

APACHE: Acute Physiology and Chronic Health Evaluation
CI: Confidence interval
CSI: Carer Strain Index
HADS: Hospital Anxiety and Depression Scale
ICU: Intensive Care Unit
InSPIRE: Intensive Care Syndrome: Promoting Independence and Return to Employment
ISI: Insomnia Severity Index
IQR: Interquartile range
LOS: Length of stay
MICE: Multiple Imputation by Chained Equations
OR: Odds ratio
SIMD: Scottish Index of Multiple Deprivation
STROBE: Strengthening the Reporting of Observational Studies in Epidemiology
