# Supplementary material for: A multicentre evaluation exploring the impact of an integrated health and social care intervention for the caregivers of ICU survivors
Source: Crit Care. 2022 May 24;26:152. doi: 10.1186/s13054-022-04014-z (PMC9128318; doi:10.1186/s13054-022-04014-z)
Supplement: Supplementary file 2 — Additional file 2: S2. Details of Hospital Anxiety and Depression Scale alongside common cut-off points. [file 13054_2022_4014_MOESM2_ESM.docx]

**S1: Information on the different outcome measures utilised**

| **Outcome measure** | Outcome descriptor | Details of scoring methodology |
| --- | --- | --- |
| **Hospital Anxiety and Depression Scale (HADS) (1)** | The HADS questionnaire contains 14 statements relating to mood, with 7 questions relating to depression and 7 to anxiety. | Scale Interpretation (scored separately for anxiety and depression):  0-7: Normal  8-10: Mild  11-14: Moderate  15-21: Severe |
| **Carer Strain Index (CSI) (2)** | The CSI which measures strain related to care provision from the caregiver perspective. There are elements related to emotional adjustment, social issues, and physical and financial strain. | Each question is given 1 point. A score of 7 or greater is the generally accepted cut off point for a high level of stress |
| **Insomnia Severity Index (ISI) (3)** | The ISI is a 7-question tool which has been validated as a screening tool for clinical insomnia. | Participants are asked to rank the severity of their sleep problems on a scale of 0 to 4 and answer 4 other questions regarding satisfaction with their sleeping patterns. The end result is a score of between 0 and 28. Guidelines for the interpretation of the ISI suggest that a score between 0 and 7 represents no clinically significant insomnia, 8 and 14 subclinical insomnia, 15 and 21 moderate clinical insomnia, and 22 and 28 severe clinical insomnia |

References

1. Zigmond, AS. Snaith, RP. The Hospital Anxiety and Depression Scale. Acta Psychiatr Scand 1983; 67:361-370.
2. Robinson, B. Validation of a Caregiver Strain Index. Journal of Gerontology 1983:38:344-338.
3. Bastien, CH. Vallieres, A. Morin, M. Validation of the Insomnia Severity Index as an outcome measure for insomnia research. Sleep Medicine 2001; 2:297-307.
