# Supplementary material for: A multicentre evaluation exploring the impact of an integrated health and social care intervention for the caregivers of ICU survivors
Source: Crit Care. 2022 May 24;26:152. doi: 10.1186/s13054-022-04014-z (PMC9128318; doi:10.1186/s13054-022-04014-z)
Supplement: Supplementary file 3 — Additional file 3: S3. Logistic regression model outcomes. [file 13054_2022_4014_MOESM3_ESM.docx]

**S4: Logistic Regression Outcome Models**

**Caregiver Anxiety**

| **Outcome measure** | **Adjusted estimate** | **p value** | **95% confidence interval** |
| --- | --- | --- | --- |
| InS:PIRE Intervention | 0.42 | 0.02 | 0.20-0.89 |
| Caregiver Relationship (Other) | 0.74 | 0.50 | 0.32-1.75 |
| Caregiver Age | 0.96 | 0.03 | 0.98-0.99 |
| Patient age at admission | 0.99 | 0.55 | 0.96-1.02 |
| Hospital LOS | 1.01 | 0.31 | 0.99-1.02 |
| Caregiver Gender (Male) | 0.81 | 0.58 | 0.38-1.71 |
| Time to follow-up | 1.07 | 0.15 | 0.98-1.17 |
| SIMD 2 | 0.72 | 0.53 | 0.26-2.03 |
| SIMD 3 | 1.37 | 0.58 | 0.45-4.11 |
| SIMD 4 | 1.22 | 0.75 | 0.36-4.05 |
| SIMD 5 | 0.77 | 0.64 | 0.25-2.36 |
| Patient mental health issues (pre ICU) | 2.80 | 0.04 | 1.06-7.40 |
| Intercept | 8.43 | 0.09 | 0.73-97.73 |

**Caregiver Depression**

| **Outcome measure** | **Adjusted estimate** | **p value** | **95% confidence interval** |
| --- | --- | --- | --- |
| InS:PIRE Intervention | 0.58 | 0.19 | 0.255-1.31 |
| Caregiver Relationship (Other) | 0.53 | 0.19 | 0.21-1.38 |
| Caregiver Age | 0.97 | 0.007 | 0.93-1.00 |
| Patient age at admission | 1.00 | 0.89 | 0.97-1.04 |
| Hospital LOS | 1.00 | 0.58 | 0.99-1.02 |
| Caregiver Gender (Male) | 0.97 | 0.94 | 0.44-2.16 |
| Time to follow-up | 1.04 | 0.29 | 0.97-0.12 |
| SIMD 2 | 0.95 | 0.93 | 0.33-2.80 |
| SIMD 3 | 1.89 | 0.30 | 0.57-6.29 |
| SIMD 4 | 1.53 | 0.53 | 0.41-5.70 |
| SIMD 5 | 0.68 | 0.62 | 0.14-3.31 |
| Patient mental health issues (pre ICU) | 3.60 | 0.008 | 1.41-9.16 |
| Intercept | 0.84 | 0.88 | 0.08-8.30 |

***Carer Strain***

| **Outcome measure** | **Adjusted estimate** | **p value** | **95% confidence interval** |
| --- | --- | --- | --- |
| InS:PIRE Intervention | 0.39 | 0.04 | 0.16-0.98 |
| Caregiver Relationship (Other) | 1.89 | 0.17 | 0.77-4.6 |
| Caregiver Age | 0.98 | 0.18 | 0.94-1.01 |
| Patient age at admission | 0.98 | 0.19 | 0.95-1.01 |
| Hospital LOS | 1.01 | 0.34 | 0.99-1.02 |
| Caregiver Gender (Male) | 0.53 | 0.15 | 0.22-1.26 |
| Time to follow-up | 1.02 | 0.60 | 0.93-1.13 |
| SIMD 2 | 1.45 | 0.51 | 0.47-4.54 |
| SIMD 3 | 1.07 | 0.92 | 0.26-4.42 |
| SIMD 4 | 1.19 | 0.81 | 0.30-4.67 |
| SIMD 5 | 0.78 | 0.73 | 0.18-3.27 |
| Patient mental health issues (pre ICU) | 3.57 | 0.01 | 1.43-8.92 |
| Intercept | 2.32 | 0.51 | 0.19-28.59 |

**Clinical Insomnia**

| **Outcome measure** | **Adjusted estimate** | **p value** | **95% confidence interval** |
| --- | --- | --- | --- |
| InS:PIRE Intervention | 0.36 | 0.009 | 0.17-0.77 |
| Caregiver Relationship (Other) | 0.69 | 0.40 | 0.29-1.65 |
| Caregiver Age | 0.98 | 0.34 | 0.95-1.02 |
| Patient age at admission | 0.99 | 0.48 | 0.96-1.02 |
| Hospital LOS | 1.01 | 0.30 | 0.99-1.02 |
| Caregiver Gender (Male) | 0.45 | 0.05 | 0.21-0.99 |
| Time to follow-up | 1.02 | 0.59 | 0.94-1.11 |
| SIMD 2 | 0.55 | 0.26 | 0.19-1.58 |
| SIMD 3 | 0.77 | 0.66 | 0.23-2.54 |
| SIMD 4 | 1.29 | 0.69 | 0.36-4.65 |
| SIMD 5 | 0.52 | 0.26 | 0.17-1.62 |
| Patient mental health issues (pre ICU) | 5.84 | >0.001 | 2.11-16.14 |
| Intercept | 6.76 | 0.10 | 0.67-67.94 |
